# Progression of anaemia during antenatal period among South African pregnant women

**DOI:** 10.4314/ahs.v22i3.10

**Published:** 2022-09

**Authors:** A M Hoque, ME Hoque, Guido Van Hal

**Affiliations:** 1 Medical Manager, Kwadabeka Community Health Centre, 21 Pitlochry Road, Westville 3629, South Africa; 2 Senior Research Associate, Management College of Southern Africa Durban, South Africa; 3 Social Epidemiology and Health Policy, University of Antwerp, Antwerp, Belgium

**Keywords:** Antenatal care, Haemoglobin, Kwadabeka, Management of anaemia, Prevalence

## Abstract

**Background:**

Anaemia in pregnancy is a known public health problem in South Africa. Maternal, perinatal morbidity and mortality are known to be associated with anaemia in pregnancy. Very little is known from literature with regards to the progression of anaemia during the antenatal period of pregnancy.

**Objectives:**

To estimate haemoglobin levels, the prevalence and determinants of anaemia at different gestational ages and to show the trend.

**Method:**

A retrospective cohort (follow-up) study was conducted using the antenatal clinic register. Prevalence rates of anaemia (haemoglobin < 11 g/dl) at different gestational ages were measured. Factors associated with anaemia were assessed using chi-square test and stepwise multivariate logistic regression analysis.

**Results:**

A total of 801 pregnant women were enrolled at the booking visit and followed-up during their antenatal period. The prevalence of anaemia at the booking visit was 37%. The prevalence of anaemia at 20, 26, 32 and 36 weeks of gestation were 36.6%, 39.6%, 39.8% and 29.2% respectively. Binary logistic regression at the booking visit showed that teenage women were 2.5 times more likely to have anaemia (OR=2.5, p=0.005) than older women. Women who booked during the first trimester were 60% less likely to have anaemia (OR= 0.40, P=0.005) at the booking visit and 62% less likely to be anaemic at 36 weeks of gestation (OR=0.38, p=0.013) compared to those who booked late for antenatal care.

**Conclusion:**

Prevalence of anaemia during pregnancy was high. Early booking for antenatal care was a predictor for lower rate of anaemia. Thus, health education strategy should be encouraged for early antenatal booking.

## Introduction

Sub-Saharan Africa (SSA) has the highest recorded rate of maternal mortality in the world.^1^ It is estimated that 20% of maternal deaths in SSA are associated with anaemia during pregnancy.^2^, ^3^ The estimated maternal mortality ratio (MMR) is estimated at 138 per 100 000 live births nationally and it is higher (180 per 100 000) for Kwazulu-Natal province (KZN) for the year 2015. This indicates that South Africa (SA) did not progress significantly towards the millennium developmental goals.^4^ The majority of maternal deaths are known to occur during labour, delivery and within 24 hours after delivery as a result of obstetric emergencies.^5^,^6^ Nearly half (>40%) of maternal deaths are found among pregnant women who are anaemic during the antenatal period.^7^ Anaemia during pregnancy is also a known risk factor for polyhydramnios, preterm birth, low birth weight deliveries, neonatal complications, neonatal Intensive Care Unit admission and neonatal deaths.^8^, ^9^ It is estimated that more than half a billion women of reproductive age (15–49 years) suffer from anaemia globally and more than a third of the pregnant women are found with anaemia worldwide in 2011, but the prevalence is higher in SA and other developing countries including SSA countries.^10^

The World Health Organization (WHO) defines anaemia as haemoglobin (Hb) concentration less than 11.0g/dl among pregnant women.^11^ The prevalence of anaemia among pregnant women is known to differ between countries and even between different geographic areas (rural versus urban) of a country. A recent study from a Durban hospital has reported a higher rate of anaemia (42.7%) at the booking (first) antenatal visit.^12^ A study conducted in Ghana has reported a similar rate of anaemia prevalence.^13^ A lower prevalence of 15.2% of anaemia at the third trimester gestational age (GA) is reported from a university-based hospital in Turkey with an association of longer hospital stay after delivery.^14^ Two earlier studies from rural settings in SA have reported different prevalence rates of 19.7% from Western Cape (2006) and 57% from KZN (2007) respectively at the booking visit.^15^,^16^

Iron deficiency is considered to be the main cause for anaemia in pregnancy. This results from the prolonged negative iron imbalance, which accounts for 50% of anaemia in general among women globally.^10^ The iron imbalance is due to poor dietary intake or absorption and increased needs for iron during pregnancy, and increased iron losses as a result of menstruation, worm infestation and infections including HIV.^2^, ^17^ Some genetic and socio-demographic and economic characteristics of women are also known to influence the distribution of anaemia and should be taken into consideration in designing preventive interventions for anaemia in pregnancy.^17^ The WHO recommends intermittent iron and folic acid supplementation for menstruating women living in settings where the prevalence of anaemia is 20% or higher and daily iron and folic acid supplementation for pregnant women as part of antenatal care in order to prevent anaemia in pregnancy.^11^ SA has implemented this strategy for all pregnant women attending public health care facilities. In SA, there is a policy to prevent and treat anaemia in pregnancy, and all pregnant women are expected to have their Hb measured at the booking ANC attendance, at 20 weeks, 26 weeks, between 28–32 weeks and at 36 weeks' gestation to identify and manage anaemia. Very little is known from SA with regards to the progression of anaemia during pregnancy and the antenatal period of pregnant women. It is, therefore, important to assess progressive haemoglobin levels of pregnant women regularly and its determinants to evaluate the treatment and preventive services of anaemia in pregnancy. Thus, the objectives of this study were to assess the prevalence of anaemia at different gestational ages of pregnant women, and to identify determinants of anaemia during the antenatal period in women receiving antenatal care at a midwife run obstetric unit in KwaZulu-Natal province (KZN), SA.

## Method and materials

### Setting and Population

The study was undertaken at Kwadabeka community health centre (KCHC), a primary health care (PHC) facility with a midwife run obstetric unit (MOU). It provides first level care of the people living in the peri-urban communities of Kwadabeka, the residence of over 133,000 black people. These communities are located within the municipal boundaries of eThekwini (Durban) district of KZN. Most of them are poor, homogenous, unemployed, living in informal (mainly) and formal type of dwellings and having a well-built cultural bond with the rural people of KZN and Eastern Cape Provinces. The residences are essentially reliant on public health facilities at KCHC. Unpublished data from the clinic shows a total of 240 000 headcounts annually with an average of 5 000 maternity-related cases (antenatal, delivery and post-natal). The annual antenatal clinic first booking attendance is about 1500 with approximately 700 annual deliveries. Maternity services at KCHC are available 24 hours a day and is run by trained and skilled midwives with other support staff. However, the routine ANC is provided as a day service between 07h00 to 16h00. Antenatal care and delivery services are rendered at KCHC following the national protocol and guidelines developed and implemented since 2002 and reviewed in 2015.^18^

### Study design

A retrospective cohort (follow-up) study was undertaken using data from antenatal clinic records between July and December 2018.

### Data sources, screening and management procedure

At the first antenatal booking visit, the midwives took down relevant histories, conducted examinations, screened pregnancy related conditions and systematically documented using a structured maternity record book and antenatal clinic register (AR).^18^ Both documents were nationally designed and implemented as standard antenatal clinic registers for SA. The AR was designed to capture data for the booking and follow up visits at 26 weeks, 32 weeks, 36 weeks and or later gestation age. Screening for anaemia, syphilis, HIV and rhesus factors using venous blood samples were undertaken as a routine procedure in the antenatal clinic. Every pregnant woman also received at least one routine obstetric ultrasound during the antenatal period. The mother's name, age, parity and the gestational age were recorded in the AR on the same day. The ages and parity were obtained from the mothers and gestational age was estimated by examination of the fundal height or from the women's last recorded menstrual period. A full blood count (FBC) was used to estimate the Hb level using the standard cyanmethemoglobin method with a COULTER HmXHaematology. Voluntary counselling and testing for HIV were offered to all pregnant mothers for inclusion in the universal anti-retroviral treatment (ART) programme on the same day. All pregnant women had an Hb measurement at the first antenatal visit, and repeated at 20 weeks, 26 weeks, 32 weeks, and 36 weeks gestational ages and recorded in the AR as follow up findings. Any women with Hb level of <10 g/dL was followed up with more frequent Hb measurements after initiating treatment and recorded in the AR. A haemoglobinometer (portable digital haemoglobinometer, HemoCue AB Angelholm, Sweden) was used, so that the result is available at the same visit. Referral criteria to hospital for anaemic pregnant women from the KCHC was as follows: Hb <6.0 g/dL, warrants an urgent transfer to a hospital on the same day, and Hb between 6.0–7.9 g/dL warrants an urgent transfer to a hospital, if symptoms of anaemia were present on the same day. If not symptomatic then they were referred to the hospital high-risk clinic within one week. If the Hb level was between 8.0 and 9.9 g/dL transfer to a hospital high-risk clinic was considered after one month of treatment with no improvement. If Hb <10 g/dL at > 36 weeks' gestational age was transferred to hospital for further ANC and delivery.

Each mother was given stocks of ferrous sulphate (200 mg daily) and folic acid (5 mg daily) doses for supplementation as a routine intervention to prevent anaemia in pregnancy. The dose of ferrous sulphate was given orally three times a day if the mother's Hb was between 8–9.9 g/dL (mild anaemia according to SA definition) and continued with folic acid 5 mg daily, and followed up for all women up to <36 weeks of pregnancy with mild anaemia with a repeat Hb after 4 weeks. The other hospital referral criteria were followed using the national protocol. 18 The documented information from the AR was extracted by one research assistant independently and entered into an excel spreadsheet.

### Data analysis

Relevant data from the Excel spreadsheet were exported into SPSS 22.0 and analysed. Anaemia in pregnancy was defined as haemoglobin (Hb) concentration <11 g/dL (WHO definition).^11^ The Hb were arbitrarily divided into four following groups: (i) levels >11 g/dL considered normal; (ii) between 10 - 10.9 g/dL as mild anaemia); (iii) between 7 - 9.9 g/dL as moderate anaemia and (iv) ≤7 g/dL as severe anaemia. The mean Hb levels were measured at different gestational age. Hb measured and recorded between 18–22 weeks gestational age (GA) reported as 20 weeks, between 23–28 weeks GA reported as 26 weeks, between 29–34 weeks GA reported as 32 weeks and between 35–38 weeks reported as 36 weeks GA for this study. GA was calculated considering the last menstrual period, an ultrasound dating scan and the symphysis-fundal height measurement. Age was categorised according to the South African Demographic Health Survey (SADHS) criteria into < 20 years (teenage), 20–34 years and 35–49 years; GA was categorised into first (0–12 weeks), second (13–27 weeks) and third trimesters (> 28 weeks) of gestation. Parity was categorised as nil-parity where index pregnancy was the first, multi- parity as having greater than one and less than five previous pregnancies and grand- multiparity as having greater than or equal to five previous pregnancies.^19^ Anaemia status of pregnant women was categorised into two mutually exclusive groups: anaemic as those with Hb < 11 g/dL and non-anaemic as those with Hb > 11 g/dL.^11^ Categorical variables were presented using frequencies and proportions. Differences in proportions for different visits were examined using Pearson's chi-squared (χ2) test. Factors associated with anaemia were assessed for both the first visit and last visit using stepwise multivariate (backward) logistic regression analysis. Factors significantly associated with anaemia were those that had a p < 0.05.

### Ethical considerations

The study protocol was approved by Umgungundlovu Health Ethics Review Board (Reference no. UHERB 015/2020). Additional written permission was obtained from the management of the KCHC to use the relevant data for the study. Secondary data was used, and hence informed consent was waived, but the extracted data were subsequently de-identified to ensure confidentiality and to protect participant's privacy.

## Results

A total of 820 pregnant women were enrolled in the AR for ANC during the study period. Nineteen (19) had incomplete data and therefore excluded from the study. Distribution of demographic and obstetric variables together with their association with anaemia at booking visit are presented in Table 1. The mean age of the pregnant women was 24.5 years with standard deviation (SD) of 6.3. The majority of them (71%) were aged between 20 and 34 years old and teenage pregnancy rate constituted 21.6%. At the booking visit, the mean GA was 21.4 weeks with SD of 7.1 weeks and one fifth of them (20%) booked at > 28 weeks. At the booking visit, the GA ranged from 4 to 40 weeks. The majority (61%) had multiparity (between 2–4 pregnancies) with more than a third (37%) with their first pregnancy.

**Table 1:** Distribution of maternal variables and prevalence of anaemia using Chi-square test at the booking visit

Variables	Frequency	Percent	% Anaemia	P value
Age group (n=793)				
< 20 years	171	21.6	20.2	0.008
20 – 34 years	559	70.5	75.4	
35+ years	63	7.9	4.4	
Average age (SD)	24.589 (SD=6.33)			
Gestation age (n=	794)			0.000
Gestational age < 13 weeks	117	14.7	16.8	
Gestational age 13 – 27 weeks	519	64.4	43.7	
Gestational age ≥ 28 weeks	158	19.9	36.7	
Mean gestational age	21 weeks (SD= 7.1 weeks)			
Parity (n= 686)				0.087
Nil parity	253	37.2	44.1	
Multiparity	419	60.7	39.0	
Grand multiparity	14	2.1	50	
HIV status (n=785)				
Positive	313	39.1	40.8	0.377
Negative	472	60.1	59.2	

The HIV status was known to only a quarter. After testing all, the prevalence of HIV turned out to be 40% among these pregnant women. The prevalence of anaemia at the booking visit was 37%. A significantly higher rate (74%) of anaemia was found among mothers aged 20–34 years and at their second trimester of GA (44%) with p< 0.05. Table 2 shows the estimation of anaemia (progressive) at different antenatal visits corresponding to different GAs. The highest prevalence (39.8%) of anaemia was estimated at 26 weeks GA and the lowest prevalence of 29.2% was at 36 weeks GA. The prevalence of severe anaemia ranged between 0.7% and 1.6% at different gestational ages. However, more than half of them were found with moderate anaemia. The overall mean Hb values are shown in Table 3. The highest Hb mean value (11.2 g/dl) was found at their booking (first) visit and lowest Hb mean value (10.2) was found at their 26 weeks GA visit. At the last visit the mean Hb value was 11.2 g/dl.

**Table 2:** Prevalence of anaemia of pregnant women at different gestational age

Variables	Booking visit		20 weeks		26 weeks		32 weeks		36 weeks	
	
Category of anaemia	Freq	%	Freq	%	Freq	%	Freq	%	Freq	%
**Severe Anaemia**	8	1.0	8	1.6	5	1.4	3	0.7	2	0.8
**Moderate** **Anaemia**	145	18.1	78	15.6	53	15.3	67	15.7	34	14.2
**Mild Anaemia**	148	18.5	97	19.4	79	22.8	95	22.3	34	14.2
**Normal**	499	62.3	317	63.4	209	60.4	261	61.2	170	70.8
**Total**	801		500		346		426		240	

**Table 3:** Mean Hb values at different gestational ages of pregnant women

	N	Mean	Std. Deviation
**Booking Hb**	801	11.25	1.75
**20 weeks Hb**	500	11.35	1.65
**26 weeks Hb**	346	10.22	2.69
**32 weeks Hb**	426	10.89	1.73
**36 weeks Hb**	240	11.29	1.58

Multiple comparison of the mean Hb values for subsequent visits showed that the overall mean values were significantly different (p<0.05) (Table 3, 4 & 5).

**Table 4:** ANOVA test results of mean Hb at booking visit

		Sum of Squares	df	Mean Square	F	Sig.
**Hb at 20** **weeks**	Between Groups	1164.761	85	13.703	27.784	.000
	Within Groups	204.186	414	.493		
	Total	1368.947	499			
**Hb at 26** **weeks**	Between Groups	251.136	36	6.976	.908	.611
	Within Groups	169.000	22	7.682		
	Total	420.136	58			
**Hb at 32** **weeks**	Between Groups	207.851	42	4.949	5.338	.000
	Within Groups	36.157	39	.927		
	Total	244.008	81			
**Hb at 36** **weeks**	Between Groups	191.462	68	2.816	1.171	.208
	Within Groups	411.243	171	2.405		
	Total	602.705	239			

**Table 5:** Multiple comparisons of Hb mean values at visits of different gestational ages using post hoc Tukey test

Visit at	Visit at	Mean Difference	Std. Error	Sig. (p value)	95% Confidence Interval of the mean difference	

					Lower Bound	Upper Bound
**Booking visit**	20 weeks GA	3.282*	.3195	.000	2.410	4.155
	26 weeks GA	4.184*	.3092	.000	3.340	5.029
	32 weeks GA	.947*	.3195	.026	.074	1.820
	36 weeks GA	-.228	.2087	.810	-.798	.342
**20 weeks** **gestation**	Booking visit	-3.28*	.3195	.000	-4.155	-2.410
	26 weeks GA	.902	.4263	.214	-.262	2.067
	32 weeks GA	-2.335*	.4338	.000	-3.520	-1.150
	36 weeks GA	-3.510*	.3600	.000	-4.494	-2.527
**26 weeks** **gestation**	Booking visit	-4.184*	.3092	.000	-5.029	-3.340
	20 weeks GA	-.902	.4263	.214	-2.067	.262
	32 weeks GA	-3.237*	.4263	.000	-4.402	-2.073
	36 weeks GA	-4.412*	.3510	.000	-5.371	-3.454
**32 weeks visit**	Booking visit	-.947*	.3195	.026	-1.820	-.074
	20 weeks GA	2.335*	.4338	.000	1.150	3.520
	26 weeks GA	3.237*	.4263	.000	2.073	4.402
	36 weeks GA	-1.175*	.3600	.010	-2.159	-.192
**36 weeks visit**	Booking visit	.2280	.2087	.810	-.342	.798
	20 weeks GA	3.510*	.3600	.000	2.527	4.494
	26 weeks GA	4.412*	.3510	.000	3.454	5.371
	32 weeks GA	1.175*	.3600	.010	.192	2.159

The significantly mean differences were found between the booking visit and the last visit, second visit and the last visit, third visit and the last visit, and 4th visit and the last visit.

Binary logistic regression was carried out to find the predictors for anaemia at the booking and last visits (presented in Tables 6 and 7). At the booking visit, teenage pregnant women (ages <20 years) were 2.5 times more likely to have anaemia (OR=2.5, p=0.005) when compared with older age. On the other hand, pregnant women at their first trimester (GA < 13 weeks) were 60% less likely to have anaemia (OR= 0.40, P=0.005) than women at the second and third trimester. It was found that GA was the only significant variable for being anaemic (OR=0.38, p=0.013) at 36 weeks GA. This meant that those pregnant women who had their first visit during the first trimester (<13 weeks) were 62% less likely to be anaemic at 36 weeks GA.

**Table 6:** Final step of back word stepwise logistic regression output at the booking visit

Variables	B	P value	Odds Ratio (OR)	95% C.I. for OR	
				Lower	Upper
Age*		.013			
Age 20–34 years	.684	.062	1.982	.967	4.064
Age < 20 years	.941	.005	2.562	1.327	4.946
Gestational age**		.000			
Second trimester	.283	.174	1.327	.882	1.996
First trimester	-.909	.005	.403	.213	.761
Constant	-1.265	.000	.282		

* >=35 years as reference group

** third trimester as reference group

**Table 7:** Binary logistic regression output (final) of anaemia at the 36 weeks gestational age.

Variables	B	P value	Odds Ratio (OR)	95% CI for OR	

				Lower	Upper
**Intercept**	0.856	0.669			
**Gravida**	-0.099	0.613	0,906	0.617	1.330
**Age ≥ 35 years** *					
**Age 20–34 years**	0.586	0.301	1.796	0.592	5.455
**Age < 20 years**	0.836	0.087	2.308	0.885	6.018
**Grand multipara** *					
**Multipara**	-1.835	0.247	0.160	0.007	3.578
**Para nil**	-1.726	0.318	0.178	0.006	5.271
**Third trimester** *					
**Second trimester**	0.000	0.999	1.000	0.605	1.651
**First trimester**	-0.967	0.013	0.380	0.177	0.817

* reference group

## Discussion

To the best of our knowledge, the present study is the pioneer to estimate the progression of anaemia during pregnancy in SA. The results confirm that the magnitude of anaemia during the antenatal period is greater than generally expected. The results of this study must be interpreted with caution as we could not follow up all booked pregnant women for antenatal care from the booking visit to 36 weeks GA. At the booking visit there was a total of 801 pregnant women and that number is found to reduce to 240 (30%) at 36 weeks GA. This is largely because of the pregnancy complications that pregnant women experienced during the antenatal period and thus referred to hospital for ANC and delivery. It is also possible that some pregnant women were lost to follow up at KCHC, as they either changed clinics or health facilities, had a spontaneous abortion or delivered prematurely. Women who had a high-risk pregnancy were also referred to higher level of health facilities (hospitals) for higher level of care. This was evident with the total numbers followed at different GA from this study. The actual rates of pregnancy complications and lost to follow-up during the antenatal period are not estimated in this study. However, it is known that approximately 70% of all pregnant women who use public health facilities in SA are estimated to require the services of a hospital and about 15% of them would require the services of a specialist obstetrician at a regional or tertiary hospital at some stage during their antenatal period.^19^ Therefore, the subsequent follow up visits at different GA among these pregnant women are likely to decrease.

The latest SADHS report (2016) suggests that a vast majority (94%) of pregnant women receive ANC from health professionals and the health care utilization by them is higher in rural areas (96%) than urban areas (92%).^19^ A similar higher utilization rate is also reported from rural KZN.^20^ The HIV prevalence among these pregnant women is 40%, similar to the rate reported from rural SA.^21^ Thus, we expect a higher attendance rate of pregnant women at KCHC representing the pregnant population of the community.

Early antenatal booking rate for ANC in this study is low (15%) during the first trimester with a mean GA at booking visit being 21.4 weeks. Most of them are found to attend late for booking visit. This relates to multiple factors such as the health seeking behaviour of the pregnant women, and the perception of health care users regarding the health care workers and the health facility. This early booking rate is lower than the rate found from a recent study at a regional hospital in an urban setting.^12^ However, the early antenatal booking rate in our study is higher than the rates found from other rural settings in KZN.^16^,^20^,^22^ Maternity care in SA including ANC is free in public health facilities. It is thus intended that pregnant women attend and take advantages of early booking for ANC in spite of the position of KCHC being in the heart of the community.^23^ The booking visit at the first trimester is reported as 28% in 1998 and is found to have improved to 47% in 2016 in SA.^19^ However, the booking visit during the first trimester in our study is low and similar to the findings from Ethiopia.^24^ It is also much lower than the rate reported from another study conducted in Ghana.^13^ Community mobilization and health education strategy at community level are thus urgently required for early booking for ANC in this community.

The teenage pregnancy rate is considered higher (21.6%) as compared to the recent reports of 18% from Durban and 16% nationally.^12^, ^19^ A similar rate of teenage pregnancy is also observed from a rural district of KZN.^16^ In general, the teenage pregnancy rate is higher in SA. This may be attributed to the change in teenagers' sexual behaviours and activities. It has been observed that girls of younger ages in SA, such as teenagers, are involved in sexual activities frequently, have sex without reliable contraceptive protection and are often the victims of forced sexual initiation that leads to a higher rate of teenage pregnancy.^25^ Teenage pregnancies are also seen to occur within the context of unstable relationships with the father of the baby, and are often unplanned or unwanted.^26^ A child support grant was introduced in SA for unemployed mothers in 1998.^23^ This is found to increase teenage pregnancy in rural areas.^27^ A higher rate of teenage pregnancy poses further problems with the sexual and reproductive health and has serious implications for the spread of sexually transmitted infections (STIs) including HIV. Some reports indicate that unplanned pregnancy among teenagers predispose to unsafe abortions and premature and preventable deaths.^25^,^26^ In our study, teenage pregnancy was found to be a risk factor (2.5 times more likely) for anaemia at the booking visit. Health education programs in school should target teenagers regarding pregnancy, their development and avoidance of teenage pregnancy.

The mean Hb level at the booking visit is higher (11.25gm/dl) than the level of diagnosing anaemia in pregnancy and the prevalence of anaemia is 37.7%. Higher rates of anaemia are found among those who presented in their second (43.7%) and third (36.7%) trimesters of GA compared to the first trimester (16.8%) (p<0.05). The rate is comparable with other recent reports from KZN and Africa.^12^,^13^,^24^ However, the prevalence rates are lower than those found from earlier reports from rural KZN.^16^,^22^ This should be seen as an improvement of management of anaemia in pregnancy for the rural people of SA. Early booking (during first trimester) for ANC has a protective effect on anaemia concurrent to findings in other studies.^15^ However, the age group of the pregnant women (between 20 – 34 years) is at higher risk of anaemia at booking visit in our study. Antenatal booking at the first trimester is found with protective effect for anaemia at > 36 weeks' GA. It is probably because of an early initiation of haematinics (Ferrous sulphate and folic acid) from first antenatal visit based on the Hb estimation and supplementation of haematinics as a routine practice for ANC in SA.^18^

The highest prevalence of anaemia was found at 26 weeks of gestation (39.6%) with the corresponding lowest mean Hb value. The encouraging findings are that there are significantly increasing mean Hb values at 32- and 36-weeks' gestation (as pregnancy advanced) and that the lowest prevalence of anaemia is at 36 weeks' gestation (29%). This could be related to the intervention undertaken by introducing haematinics from the first antenatal visit and the known physiological changes.^28^–^31^ The step-by-step management of anaemia adopted at KCHC according to the national protocol using ferrous sulphate and folic acid might have reduced anaemia at 36 weeks' gestation. This is supported by the recent findings that overall, 90% of women are found with taking iron tablets during pregnancy in SA.^19^ A higher rate of reduction of anaemia is expected to avert the negative effects on mothers and new-borns.

The prevalence of anaemia at 32- and 36-weeks' gestation in our study are still higher compared with the rate reported from other studies.^14^, ^24^ This could be related to different socio-economic environments of the study population. Our study subjects are predominantly black South African pregnant women with low socio-economic status. Variations in mean Hb levels and prevalence rates of anaemia at the booking visit and subsequent follow-up visits during antenatal period require an establishment of reference levels for pregnant populations in SA. This is considered difficult logistically for different geographic locations, settings, diversity of the population (racial and others) and the geographical nature of SA, for example people living at high altitudes like mountains. It has been reported that factors such as high altitude, genetics and nutrition play an important role on the Hb levels in the general and pregnant population.^32^–^34^ However, our study can be considered a step forward to set up such reference for rural KZN in SA.

A number of normal and abnormal changes in anatomy, physiology and biochemistry are known to occur during pregnancy which are likely to impact on maternal health.^28^ The known common problem among them is the expansion of plasma volume (25 -80%) between pre pregnancy, the normal physiological changes in pregnancy during the second trimester and the middle of the third trimester of pregnancy but the corresponding red cell volume expands by only 18–25%. This leads to haemodilution that leads to “physiological anaemia of pregnancy”.^28^–^31^ However, there is a rise in total circulating haemoglobin directly related to the increase in red cell mass.^30^, ^31^ This is known to induce a modest decrease in Hb levels during pregnancy. We have observed a similar trend of Hb values in our study (Table 3) that the lowest mean Hb value is at 26 weeks GA with the highest corresponding prevalence of anaemia. Based on these physiological changes, the Centres for Disease Control and Prevention suggest that anaemia in pregnancy should be defined as haemoglobin levels < 11 g/dL in the first and third trimesters and less than 10.5 g/dL in the second trimester.^35^ Therefore, previous report suggests that the best time to investigate any risk factors associated with anaemia during pregnancy is before 20 weeks of gestation.^36^

In our study, the mean GA at the booking visit is late of 21 weeks. It is known that fluctuations in Hb levels occur by GA of pregnancy (measured by trimester) as a result of maternal and foetal physiological demands. It therefore, suggests that a 1.0 g/dL decrease takes place between different GA as the first and third trimester of pregnancy, with a further reduction of Hb concentrations of 0.5 g/dL in the second trimester.^37^ A similar trend of mean Hb values are obtained in our study (table 3). However, it is not clear what the contribution of physiological anaemia in our study population is. Although we defined anaemia according to WHO recommendations for practical reasons, we did not consider trimester adjusted Hb cut-off levels. There is a known factor of progressive increase in the Packed Cell Volume from the first to the third trimester GA and a progressive decrease of Hb with increasing maternal ages and numbers of previous pregnancies.^35^ Therefore, GA at the booking visit of our study is found to protect against anaemia at both the booking and 36 weeks' gestation visits and is similar to other finding.^8^

Anaemia in women is known as a result of iron deficiency in most cases. Deficiency of iron in pregnancy is most likely due to inadequate and insufficient storage of iron to meet the increased requirements of pregnancy. The known risk factors in less developed countries are intestinal parasitic infections, HIV infection and haemoglobinopathies which contribute to the higher rates of anaemia during pregnancy and in general.^17^, ^38^–^42^ Teenage pregnancies, pregnancy among elderly women, women with very low body mass index (BMI) and grand multiparity are known as other intermediate obstetric causes of anaemia.^43^–^45^ The demographic, geographical, socio-economic, religious, cultural factors of pregnant women as well as access to adequate ANC services are other known relative contributions to anaemia in pregnancy.^46^ There are different views on the universal approach to prophylactic iron therapy during pregnancy, e. g. that it might neglect underlying untreated diseases and thus may fail to correct anaemia.^47^ Some of the above factors are prevalent in our study population, such as a higher rate of teenage pregnancy, HIV infection thus might have contributed to the higher prevalence of anaemia in this population. The result confirms that the progression of anaemia during antenatal period is a common health problem in our setting. However, there is a declining trend of anaemia from the booking visit to 36 weeks GA in our population.

## Study Limitations

The main limitation of this study is the retrospective design. However, the present study is conducted in a first level of health facility situated in the community with a defined population and we did not exclude any high-risk patients at the booking visit. The pregnant women with obstetric and medical complications during different antenatal visits were referred to hospitals which resulted in low-risk pregnancies followed-up until the 36 weeks GA and subsequently resulted in a high loss to follow up rate. Therefore, results should be interpreted based on its limitations.

## Conclusion

The prevalence of anaemia at the booking, 20 weeks, 26 weeks, 32 weeks and 36 weeks gestations is are high and a major health problem in this setting at the study site in SA. A declining trend of anaemia is observed as pregnancy advanced. There is a need to strengthen our preventive and promotive healthcare system to ensure early booking for ANC and definitive diagnosis so that appropriate treatment can be provided in early pregnancy. Further studies to identify the causes of anaemia in pregnancy especially diet, personal habits, practices and infections are recommended.
